# Adrenal Collision Tumor Composed of Pheochromocytoma and Diffuse Large B-Cell Lymphoma: A Case Report

**Published:** 2018-10-01

**Authors:** Atieh Khorsand, Fatemeh Khatami, Salma Sefidbakht, Hiva Saffar, Alireza Sadeghipour, Seyed Mohammad Tavangar

**Affiliations:** 1 Department of Pathology, Shariati Hospital, Tehran University of Medical Sciences, Tehran, Iran; 2 Chronic Diseases Research Center, Endocrinology and Metabolism Population Sciences Institute, Tehran University of Medical Sciences, Tehran, Iran; 3 Department of Pathology, Rasool-e-Akram Hospital, Iran University of Medical Sciences, Tehran, Iran

**Keywords:** Collision tumor, Diffuse large B-cell lymphoma, Pheochromocytoma

## Abstract

Adrenal involvement in the course of malignant lymphoma occurs in about 4% of patients, but primary adrenal lymphoma (PAL) is extremely rare. To the best of our knowledge, only one case study reported the combination of PAL with pheochromocytoma. In the current study, we present the second case who was a 63-year-old man admitted to our hospital with hematuria and abdominal discomfort. Ultrasound imaging indicated the presence of a lesion, 5×4×3 cm in size, in the upper pole of his right kidney. Histopathologic study confirmed a collision tumor composed of pheochromocytoma and diffuse large B-cell lymphoma (DLBCL).

## Introduction

 Primary lymphomas of endocrine glands are extremely rare and usually involve thyroid gland and less commonly adrenal glands^   1 ^. Pheochromocytomas as the neoplastic transformation of chromaffin cells of sympatoadrenal system are also rare and usually pure neoplasms ^2^^-^^4^ . Coexistence of PAL and pehochromocytoma is very rare and we describe a unique presentation of this association^   5 ^.

## Case presentation

 A 63-year-old man with three different episodes of renal colic and painful hematuria was referred to our hospital. He had no specific medical history or hormonal symptoms. Physical examination was unremarkable and he had normal vital signs. In paraclinical investigations, ultrasound imaging revealed a mass in upper pole of right kidney. Pheochromocytoma workup was negative. The patient underwent right adrenalectomy. The submitted sample for pathologic study consisted of multiple fragmented irregular tumoral tissues, weighting 95 gram measuring totally 5×4×3 cm.

Microscopic examinations showed two separated areas, one component was composed of nests and sheets of neoplastic large polyhedral cells in a vascularized stroma .The cells had round to oval moderately pleomorphic nuclei, distinct nucleoli and basophilic cytoplasm.

**Figure 1:**
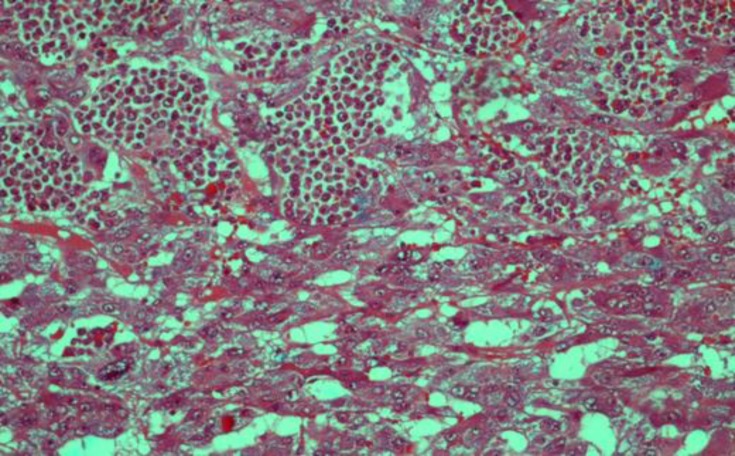
(H&E, times 250) Boundary between pheochromocytoma and Lymphoma

**Figure2:**
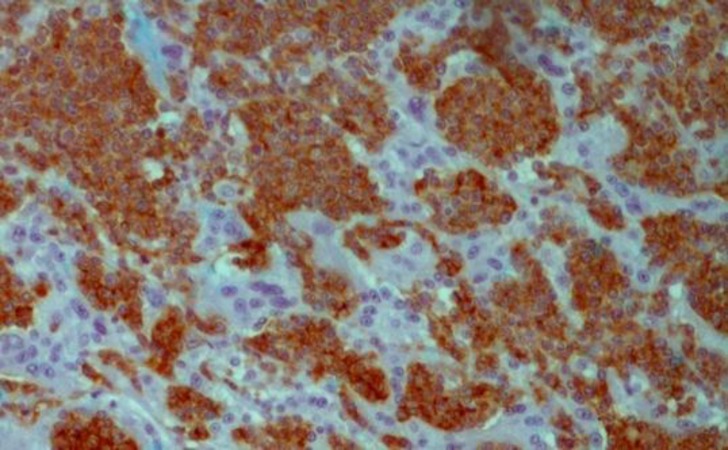
IHC (×400), Lymphoma cells show positive reaction for CD20 antigen

**Figure 3:**
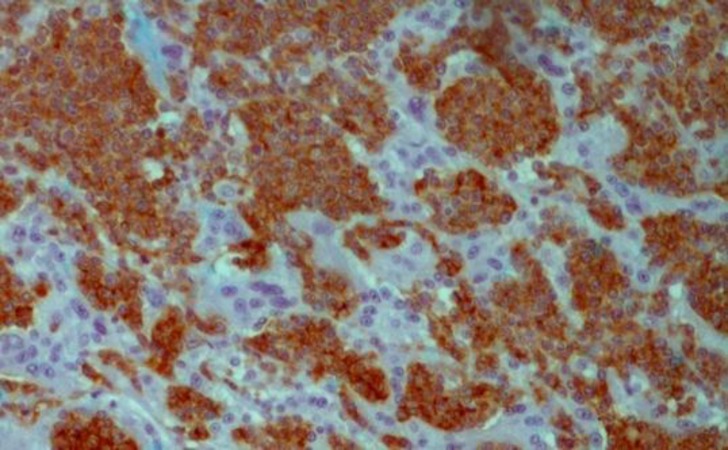
IHC (×400), The pheochromocytoma component shows strong immune reactive for chromogranin antibody

 In many areas, tumoral cells were invaded and trapped by the second population of small round tumoral cells with high N/C ratio, vesicular mildly pleomorphic nuclei and thick irregular nuclear membrane. A lot of mitotic figures were identified. Immunohistochemical (IHC) staining signified these cells as positive cells for CD20 and BCL2, and negative for CK, CD3, BCL-6, MUM-1 and CD10 (Figure 2). Ki67staining showed proliferative activity in about 60% of tumor cells. Histologically, the tumor approximately consisted of 80% typical pheochromocytomas and 20% diffuse large B-cell lymphoma (DLBCL) (Figure 1). IHC studies in the pheochromocytoma component showed positivity of tumor cells for synaptophysin and chromogranin (Figure 3).

## Discussion

 Adrenal collision tumors (ACTs) by definition show the presence of two independent tumors in one adrenal gland. Co-occurrence of pheochromocytoma with neuroblastoma, ganglioneuroblastoma, ganglioneuroma or malignant peripheral nerve sheath tumor (MPNST) has been reported several times during the past 70 years  ^6^^-^^9^ .

In one review, Brian R Untch et al. showed that all ACLs had one component of primary adrenal tumors and the second component in most cases was metastasis^   10 ^.The latter tumoral component can be lymphoma^   11 ^.

PAL is usually represented with bilateral large masses, sometimes accompanied by adrenal insufficiency, most frequently affecting older men^   12 ^. The lymphoma part of our case was DLBCL which can arise in almost any part of the body and the first manifestation of this cancer is typically a rapidly growing mass, sometimes associated with B symptoms—fever, weight loss, and night sweats^   13 ^. Our case showed none of the above-mentioned symptoms.

On the other hand, one of primary adrenal tumors that can be seen in ACLs is pheochromocytoma which secretes catecholamines, and therefore headache, sweating palpitations and hypertension are the most common symptoms. Our case had none of these symptoms and biochemically was negative for pheochromocytoma markers. Pheochromocytomas with negative urine and serum markers are not uncommon ^14^^,^^15^ . Heavner et al. showed some differences between marker-negative and marker positive patients, including different mean BMI and presentation signs (more vertigo/dizziness in marker-negative patients)^   14 ^.

The majority of pheochromocytomas are benign and curable by resection. However, differentiation between benign and malignant pheochromocytoma is one of the most challenging areas in endocrine pathology. Application of pheochromocytoma of the Adrenal Gland Scaled Score (PASS) is one the recent attempts for distinguishing pheochromosytomas with malignant potential, which showed great inter-and intra-observer variation^   16 ^. Immunohistochemical study is a great method in detection of different aspects of diagnosis and treatment ^17^^-^^23^ . In one study, Saffar et al. examined 55 cases of pheochromocytoma and determined the expression of galectin-3, COX-2, and nm-23 among them^   24 ^. They showed that the presence of these markers along with tumor size and tumor necrosis can be a reasonable predictor of this disease outcome.

In another study, Haji Amousha, et al. used the pituitary tumor-transforming gene (PTTG1) to predict malignant behavior of pheochromocytomas^   25 ^. They showed that this IHC marker had 100% specificity and 100% PPV for malignant behavior prediction. Ki67,c-erbB-2 and c-kitantigens were also used as predictor markers of tumor behavior in another study by Tavangar et al^   19 ^. Their study demonstrated that these markers could be used to determine the behavior of tumors. The pheochromocytoma part of our case had no metastasis and showed benign behavior.

## CONCLUSION

 The results of the study highlight the need to increase awareness of rare entities such as ACLs for proper diagnosis of adrenal masses in paraclinical investigations which is critical for staging and treatment.
